# Organ-specific requirements for Hdac1 in liver and pancreas formation

**DOI:** 10.1016/j.ydbio.2008.06.040

**Published:** 2008-10-15

**Authors:** Emily S. Noël, Antonio Casal-Sueiro, Elisabeth Busch-Nentwich, Heather Verkade, P. Duc Si Dong, Derek L. Stemple, Elke A. Ober

**Affiliations:** a National Institute for Medical Research, Division of Developmental Biology, The Ridgeway, Mill Hill, London, NW7 1AA, UK; b Wellcome Trust Sanger Institute, Wellcome Trust Genome, Campus Hinxton, Cambridge, CB10 1SA, UK; c Department of Biochemistry and Biophysics, Programs in Developmental Biology, Genetics and Human Genetics, Liver Center, University of California, San Francisco, CA 94158, USA

**Keywords:** Hdac1, Liver, Pancreas, Chromatin remodelling, Zebrafish

## Abstract

Liver, pancreas and lung originate from the presumptive foregut in temporal and spatial proximity. This requires precisely orchestrated transcriptional activation and repression of organ-specific gene expression within the same cell. Here, we show distinct roles for the chromatin remodelling factor and transcriptional repressor Histone deacetylase 1 (Hdac1) in endodermal organogenesis in zebrafish. Loss of Hdac1 causes defects in timely liver specification and in subsequent differentiation. Mosaic analyses reveal a cell-autonomous requirement for *hdac1* within the hepatic endoderm. Our studies further reveal specific functions for Hdac1 in pancreas development. Loss of *hdac1* causes the formation of ectopic endocrine clusters anteriorly to the main islet, as well as defects in exocrine pancreas specification and differentiation. In addition, we observe defects in extrahepatopancreatic duct formation and morphogenesis. Finally, loss of *hdac1* results in an expansion of the foregut endoderm in the domain from which the liver and pancreas originate.

Our genetic studies demonstrate that Hdac1 is crucial for regulating distinct steps in endodermal organogenesis. This suggests a model in which Hdac1 may directly or indirectly restrict foregut fates while promoting hepatic and exocrine pancreatic specification and differentiation, as well as pancreatic endocrine islet morphogenesis. These findings establish zebrafish as a tractable system to investigate chromatin remodelling factor functions in controlling gene expression programmes in vertebrate endodermal organogenesis.

## Introduction

The development of a multi-organ structure requires the temporally and spatially coordinated regulation of gene expression. Neighboring groups of cells, which initially share a common gene expression programme, will adopt different fates by expressing different sets of genes. This is realised by actively regulated initiation and termination of transcription, which in turn depends on the presence of specific activating and repressing transcription factors, and importantly on their ability to access regulatory gene elements. It has been shown that chromatin modifications, such as methylation or acetylation are central to regulating gene expression (Jenuwein and Allis, 2001; Kouzarides, 2007). DNA methylation represents a stable and heritable mechanism for epigenetic silencing of transcription (Goll and Bestor, 2005). In contrast, histone acetylation mediated by Histone acetyltransferases prevents chromatin condensation, thus allowing transcriptional activation. Conversely, removal of acetyl groups leads to chromatin compaction resulting in transcriptional repression. De-acetylation is mediated by Histone deacetylases, which are grouped into four classes based on their homology to yeast: Hdac1, 2, 3 and 8 (class I), Hdac4, 5, 6, 7, 9 and 10 (class II), Sir2-like Hdac (class III) and Hdac 11. The latter shares similarity with class I and II enzymes (de Ruijter et al., 2003).

Although the importance of Hdacs in gene regulation is well established, and their specific roles in differentiation of embryonic stem cells, as well as hepatic and pancreatic cancer are emerging (Glozak and Seto, 2007), their specific roles in embryonic development are still poorly understood. For instance, in mice, depletion of Hdac1 in the entire embryo leads to widespread proliferation defects during gastrulation and early lethality that are at least partly due to up-regulation of the cell-cycle inhibitor p21 (Lagger et al., 2002). In contrast, because of maternal contribution zebrafish *hdac1* mutant embryos pass through gastrulation exhibiting mild patterning defects in a subset of tissues, but without severe early morphological defects (Nambiar and Henion, 2004; Nambiar et al., 2007). Thus, Hdac1 dependent processes occurring at later stages of embryonic development can be examined, such as neurogenesis, eye or fin development (Cunliffe, 2004; Stadler et al., 2005; Yamaguchi et al., 2005). Hence, zebrafish is a highly suitable model for elucidating the role(s) of Hdac1 in endodermal organogenesis.

The endodermal organ system consists of the digestive tract and its accessory organs — liver, pancreas and lungs or the inner lining of the swim bladder, in mammals and zebrafish, respectively. The organs arise in close temporal and spatial proximity from the foregut endoderm (Grapin-Botton, 2005). The foregut and the organs derived from it express different combinations of transcription factors, such as members of the Gata, FoxA and Hnf families, which play different roles in organ specification and differentiation (Duncan, 2000; Kaestner, 2005; Zaret, 2002). In zebrafish, the endocrine pancreas is the first to develop from the dorsal side, by aggregation of the endocrine islet (Argenton et al., 1999; Biemar et al., 2001). This is closely followed by specification of the liver on the ventral side, anterior to the endocrine islet (Field et al., 2003b). Hepatoblasts, the liver precursor cells, express the transcription factors Hhex and Prox1 (Ober et al., 2003; Wallace and Pack, 2003) and differentiate into mature hepatocytes and biliary cells. Liver specification requires the interaction between the foregut endoderm and the neighboring lateral plate mesoderm (Grapin-Botton, 2005; Zaret, 2002). The LPM releases hepatoblast-inducing factors that include Fgf, Bmp and Wnt family of signalling molecules (Grapin-Botton, 2005; Ober et al., 2006). Next, a second, exocrine pancreatic primordium arises from the ventral foregut endoderm close to the forming hepatic bud. The exocrine and endocrine primordium fuse and ultimately become connected by a common extrahepatopancreatic duct (Field et al., 2003a; Wallace and Pack, 2003; Yee et al., 2001). A number of transcription factors have been implicated in specific endocrine or exocrine development, such as NeuroD1 and Neurogenin3, and Hes1 and Ptf1a, respectively (Cano et al., 2007). Similar to the interactions required during hepatic development, the mesoderm adjacent to the presumptive pancreatic tissues releases signals such Retinoic acid (RA) and members of the Fgf and Bmp families of secreted molecules (Cano et al., 2007; Grapin-Botton, 2005) that regulate pancreatic organogenesis. In pancreatic and hepatic development, the respective inductive signalling cascades regulate transcription of genes specific for the induction and differentiation of each organ. Investigating the roles of factors controlling the accessibility of regulatory elements mediating this transcription, will further our understanding of how organ-specific gene expression programmes are realised.

Here, we describe the mutant line *s436*, a novel allele of *hdac1* in zebrafish, which despite its broad expression displays distinct defects in endodermal organogenesis. In *hdac1* mutants hepatic and exocrine pancreatic specification and differentiation are severely affected. This is accompanied by defects in extrahepatopancreatic duct formation and an expansion of foregut tissue. Moreover in *hdac1* mutants, we observe ectopic endocrine islet formation. Our genetic studies reveal that Hdac1 is required for the establishment of hepatic and exocrine pancreatic cell fates within the foregut, which occurs at the expense of the tissue forming the alimentary canal, suggesting a model in which an epigenetic enzyme mediates a fate switch at the organ level.

Taken together, we present very different yet crucial roles for the chromatin modification factor *hdac1* in hepatic, pancreatic and foregut organogenesis in the zebrafish embryo.

## Materials and methods

### Fish stocks

Adult zebrafish and embryos were raised according to standard laboratory conditions (Westerfield, 2000). The following strains were used: *hdac1*^*s436*^ (referred to as *hdac1*), *hdac1*^*hi1618*^ (Golling et al., 2002), *Tg(gutGFP)*^*s854*^ (Field et al., 2003b), *Tg(lfabp:dsRed*; *elastaseA:GFP)* (Dong et al., 2007), *casanova*^*ta56*^ (Kikuchi et al., 2001), and wild type lines SJD and LonTuploff.

### Genetic mapping and positional cloning

A mapping strain was created by crossing a *hdac1*^*s436*^/*Tg(gutGFP)*^*s854*^ female to a wild type SJD male. Bulk segregant analysis and fine mapping linkage analysis were performed on sibling and mutant embryos using SSLP primers (MWG Biotech).

Complementation studies for *hdac1* were carried out crossing heterozygous fish for the *hdac1*^*hi1618*^ allele and the *hdac1*^*s436*^ allele.

Two independent cDNA preparations were made from mutant and wild type embryos using Superscript First Strand Synthesis System (Invitrogen), and four independent sequencing reactions were carried out per cDNA preparation.

### Western blot analysis

Embryos were deyolked in chilled PBST (PBS, 0.1% Tween-20) and pools of 25 embryos for each genotype and stage frozen at − 80 °C. Embryos were homogenised in SDS-Gel-loading buffer (100 mM Tris pH 6.8, 4% SDS, 0.2% Bromophenol blue, 20% glycerol, 200 mM β-mercaptoethanol) and heated at 98 °C to extract proteins. Samples were spun at 4 °C for 5 min and the supernatant recovered. Protein samples were normalized using Coomassie Blue staining. Extracted proteins were run on a 12% SDS gel, and transferred to a PVDF membrane (BioRad). Membranes were blocked with 5% nonfat powdered milk in PBST and incubated with anti-hyperacetylated histone H4 antibody (1:1000, Upstate), or anti-β-actin antibody (1:1000, Sigma) overnight at 4 °C. Membranes were washed in blocking solution, and incubated with anti-rabbit HRP IgG antibody (1:5000, BioRad) for 30 min. After washing with blocking solution, blots were visualised using enhanced chemiluminescence (Amersham). Experiments were repeated with samples homogenised in RIPA buffer and protein content determined using Bradford assay prior to gel loading. 0.44 μg of protein was loaded per sample.

### Immunohistochemistry

Immunostainings were carried out as previously described (Ober et al., 2006), using monoclonal antibodies against 2F11 (Dong et al., 2007; gift from Julian Lewis, Cancer UK, 1:1000), Islet1/2 (Developmental Studies Hybridoma Bank, 1:15), phospho-Histone H3 (Abcam, 1:500), and rabbit polyclonal antibodies against Prox1 (Chemicon, 1:1000) and GFP (Torrey Pines Biolabs, 1:500). Fluorophore-conjugated secondary antibodies were obtained from Jackson Laboratories (USA). Fluorophore-conjugated phalloidin (Sigma) was used at 1:50. Embryos were mounted in 4% low-melting-point agarose/1% gelatine, 130–140 μm sections were prepared using a Leica Vibratome and visualised using a Leica DMRXE SP1 or a Zeiss LSM5 Pascal Exciter confocal microscope. Images were processed using Volocity image analysis software (Improvision).

### Quantification of endodermal cell number

Immunohistochemistry was carried out as described above. 90 μm transverse sections were prepared, and optical stacks were taken using a Zeiss LSM5 Pascal Exciter confocal microscope throughout the organ-forming region. For each stack, cell numbers were determined in three separate optical slices. The numbers of total endodermal nuclei, Prox1-positive hepatoblast nuclei, and Prox1-positive exocrine pancreatic nuclei were counted manually, using Volocity image analysis software for visualisation.

### In situ hybridisation

Whole-mount mRNA in situ hybridisation was performed as described (Alexander et al., 1998). Embryos older than 24 hpf were treated with 0.2 mM 1-phenyl-2-thiourea (PTU) in egg water to inhibit melanin production.

Fluorescent in situ hybridisations were carried out using the TSA Plus Fluorescence palette system (Perkin-Elmer). Anti-digoxygenin-HRP (Roche) and anti-fluorescein-HRP (Perkin-Elmer) were used at 1:1000; tyramide-labelled substrate was used at 1:50. The following probes were used: *ceruloplasmin* (Korzh et al., 2001), *claudin15* (Bagnat et al., 2007), *foxA1* (Odenthal and Nusslein-Volhard, 1998), *hdac1* (Pillai et al., 2004), *her5* (Muller et al., 1996), *hhex* (Ho et al., 1999), *insulin* (Milewski et al., 1998), *meis3* (Sagerstrom et al., 2001), *pes* (Allende et al., 1996), *ptf1a* (Lin et al., 2004), *prt/wnt2bb* (Ober et al., 2006), *trypsin* (Biemar et al., 2001).

### Transplantation

Transplants were performed as previously described (Stafford et al., 2006); *cas* sense RNA was generated using the mMESSAGE mMACHINE SP6 kit (Ambion) from the pCS2*cas* construct (Aoki et al., 2002) and 100–200 pg were injected into 1-cell stage wild type or *Tg(gutGFP)*^*s854*^ embryos. *Casanova* morpholino sequence: 5′-CAGGGAGCATCCGGTCGAGATACAT-3′ (Dickmeis et al., 2001). Mosaic embryos were raised in Danieau buffer with Penicillin–Streptomycin (0.1%; Sigma) and fixed in 4% paraformaldehyde (PFA).

### TSA treatment

Trichostatin A (TSA, Invivogen) was resuspended in DMSO to 1 mg/ml, and diluted in egg water to concentrations between 200 nM and 1200 nM. Embryos were incubated at 28 °C in TSA (control embryos were treated with equal amounts of DMSO) in a shaking incubator.

### Labelling for apoptosis

Embryos were fixed in 4% PFA and dehydrated in MeOH. After rehydration, embryos were treated with Proteinase K, and immunolabelling was performed as previously described. Apoptotoic cells were detected by terminal deoxynucleotidyl transferase-mediated dUTP nick-end labelling (TUNEL), using an In Situ Cell Death Detection Kit (Roche).

## Results

### Hdac1 plays multiple roles in liver and pancreas formation

**Fig. 1:**
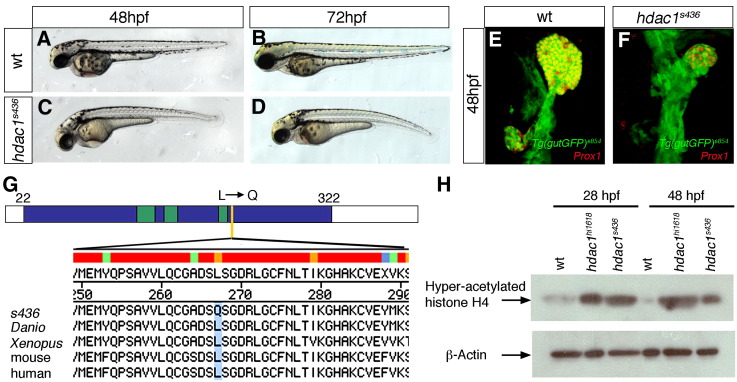
Mutant line *s463* encodes a novel *hdac1* allele. Lateral brightfield views of sibling (A, B) and *hdac1*^*s436*^ mutant (C, D) embryos at 48 hpf and 72 hpf, anterior to the left. (E, F) Projections of confocal stacks showing ventral views of *Tg(gutGFP)*^*s854*^, anterior to the top. (E) Wild type embryo at 48 hpf, expressing Prox1 (red) in the liver and exocrine pancreas. (F) *s436* mutant exhibits a reduced liver and absent exocrine pancreas at 48 hpf. (G) *s436* encodes a novel allele of Hdac1. A T to A base pair change at position 800 results in a leucine to glutamine transition at position 267. Schematic representation of the predicted zebrafish Hdac1 derived from alignment of vertebrate sequences; catalytic domain is depicted in blue, HDAC superfamily signature sequences in green, *s436* lesion in yellow. Protein alignments with amino acid transition shown in blue. (H) Western blot showing increased levels of acetylated histone H4 in both *hdac1*^*hi1618*^ and *hdac1*^*s436*^ mutant embryos at 28 hpf and 48 hpf. β-actin levels were used as loading control.

In a forward genetic screen designed to specifically identify genes required for endodermal organogenesis in zebrafish (E.A.O., H.V., H.A. Field, P.D.S.D., P. Aanstad, T. Sakaguchi, M. Bagnat, C. Munson,W-S. Chung., C.H. Shin., S. Curado, R. Anderson, J. Frantsve, D. Beis, T. Bartman and D.Y.R. Stainier., unpublished observations), we isolated the mutant line *s436* exhibiting distinct defects in liver and pancreas development. At 48 hours post-fertilisation (hpf) in wild type, both organs are established and functional differentiation is in process. In contrast, in *s436* the liver and pancreas are not only small, but additionally exhibit morphogenesis defects (Figs. 1E, F). To understand the molecular nature of the *s436* phenotype, we mapped the affected genetic locus in the *s436* mutant. A set of simple sequence length polymorphism (SSLP) markers for recombination mapping was used to place the mutant locus between the two markers z15237 and z22532 on linkage group 19. Analysis of 560 meiosis revealed 1 and 9 recombination events for z15237 and z22532, respectively. The region these two markers encompass contains approximately 40 genes, including *hdac1*. *hdac1*^*hi1618*^ mutant embryos exhibit similar body phenotypes, such as heart, fin and body shape defects, as observed in *s436* mutant embryos (Golling et al., 2002; Nambiar et al., 2007; Stadler et al., 2005; Yamaguchi et al., 2005). Moreover, *s436* failed to complement the known *hdac1*^*hi1618*^ allele (Golling et al., 2002), suggesting that *s436* represents a novel allele of *hdac1*. Sequencing of *hdac1* from both wild type and *s436* mutant embryos identified a T to A point mutation at position 800 (Fig. 1G), resulting in the replacement of a conserved leucine with a glutamine at amino acid 267. Hdac1 is highly conserved among vertebrates, with 96% similarity between the catalytic domain of zebrafish and human (aa22–322). The Leu 267, corresponding to Leu 266 in humans, is located in the binding pocket of the catalytic active site of Hdac1 (Fig. 1G; (Finnin et al., 1999). The position of the point mutation in *hdac1*^*s436*^ suggests that the catalytic deacetylase function is impaired. To determine changes of deacetylase activity in *s436* mutants, we compared the levels of histone acetylation in wild type and mutant embryos. Western blot analysis using an anti-hyperacetylated histone H4 antibody, revealed increased levels of histone acetylation in *s436* embryos when compared to levels in wild type embryos at 28 and 48 hpf (Fig. 1H). Levels of histone H4 acetylation in *s436* mutants were similar to the levels observed in *hdac1*^*hi1618*^ mutants at these stages. Moreover, the expression of four different genes, expressed in the forming hepatoblasts, differentiating liver, early exocrine pancreas and pancreatic β-cells, was compared between *hdac1*^*s436*^ mutants and mutants for the insertional allele, *hdac1*^*hi1618*^ (progeny of F1 carriers in the *Tg(gutGFP)*^*s854*^ background). This analysis revealed qualitatively highly similar phenotypes of differing penetrance for the examined organs (Supplemental Table 1; Figs. 3 and 7). Notably, phenotypic analysis of the *hdac1*^*hi1618*^ mutation in different genetic backgrounds has revealed variable phenotypic severity (Pillai et al., 2004). Taken together, these data confirm that *s436* encodes a loss-of-function allele of *hdac1*, possibly a null allele, hereafter referred to as *hdac1*.

### Requirement of Hdac1 for timely liver specification and differentiation

**Fig. 2:**
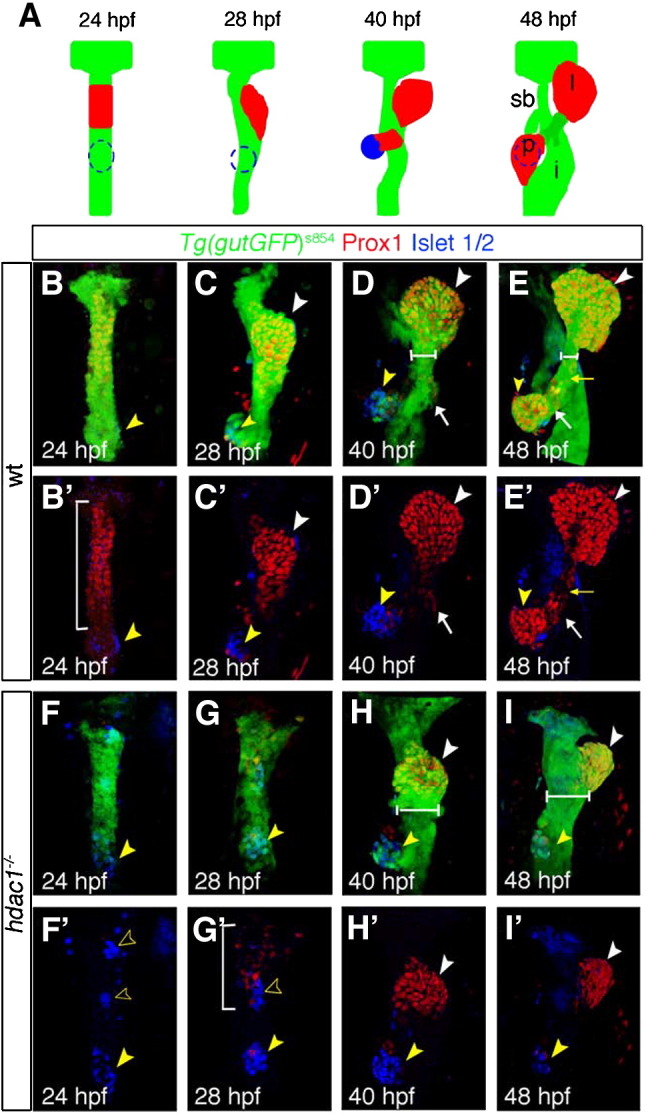
Specific requirements for Hdac1 in hepatic and pancreatic development. (A) Schematic depicting stages of endodermal organogenesis in wild type. Endoderm in green, Prox1-positive liver and exocrine pancreas in red, and endocrine pancreas in blue. l, liver; p, pancreas; dotted line, endocrine pancreas; sb, swim bladder; and i, intestinal bulb. (B–I′) Time course analysis comparing endodermal organogenesis of wild type siblings and *hdac1* mutants, using the *Tg(gutGFP)*^*s854*^ line stained for Prox1(red) and Islet 1/2 (blue). Projection of confocal stacks showing ventral views of the *Tg(gutGFP)*^*s854*^ line expressing GFP throughout the endoderm (green), anterior to top. (B–E′), Wild type siblings, (F–I′), *hdac1* mutants. (B, B′) At 24 hpf Prox1-expressing hepatoblasts are present throughout the organ-forming region of the endodermal rod (bracket). The endocrine pancreatic islet, stained with Islet 1/2, is situated posteriorly in the organ-forming region (yellow arrowhead). (C, C′) By 28 hpf the Prox1-positive hepatoblasts aggregate on the left side of the endodermal rod (white arrowhead) forming the liver bud. The exocrine pancreas expresses Prox1 at 40 hpf (arrow, D, D′) and 48 hpf; the liver and ventral pancreas are connected to the adjacent digestive tract by the extrahepatopancreatic duct (yellow arrow, E, E′). (F, F′) *hdac1* mutants lack Prox1 expression at 24 hpf. The endocrine islet is present, although anterior groups of endocrine cells have formed (empty arrowheads). By 28 hpf, Prox1 expression is initiated in hepatoblasts (bracket, G, G′) which subsequently aggregate to form a medial liver bud by 40 hpf (white arrowhead, H, H′). By 48 hpf, in *hdac1* mutants, the liver bud is located asymmetrically on the left side of the digestive tract (white arrowhead, I, I′). Exocrine pancreas and swim bladder fail to form at this time. Additionally, the width of the digestive tract is greater in *hdac1* mutants than in wild type embryos once liver bud outgrowth has been initiated (compare horizontal bars, D, E with H, I).

**Fig. 3:**
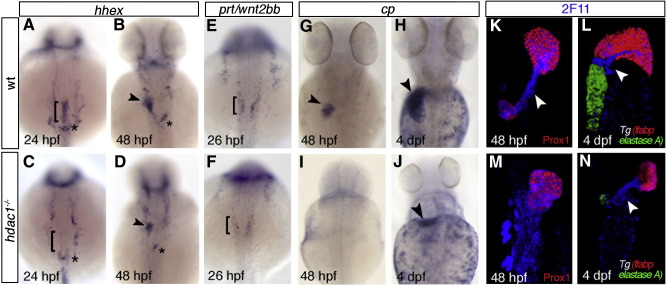
Hdac1 is required for timely liver specification and differentiation. (A–J) In situ hybridisation analyses of *hhex*, *prt/wnt2bb* and *cp* expression in sibling and *hdac1* mutant embryos; dorsal views, anterior to the top. (A, B) Wild type embryos express *hhex* in hepatoblasts at 24 hpf (bracket, A) and 48 hpf (arrowhead, B). Additionally, *hhex* is expressed in pancreatic tissue (asterisk). (C) *hdac1* mutants lack hepatic *hhex*, though retain pancreatic expression at 24 hpf. At 48 hpf *hhex* expression is present in the liver of *hdac1* mutants, although in a reduced domain (arrowhead, D). (E, F) Wild type embryos express *prt/wnt2bb* in the LPM at 26 hpf (bracket, E), however *hdac1* mutants show reduced *prt/wnt2bb* expression (bracket, F). (G–J) *cp* is expressed in differentiating hepatocytes at 48 hpf and 4 dpf in wild type embryos (arrowheads, G, H). *hdac1* mutants lack *cp* expression at 48 hpf (I); a subset of embryos express *cp* at 4 dpf (arrowhead, J). (K–N) Ventral projection of confocal stacks. Analysis of *Tg(gutGFP)*^*s854*^ embryos (K, M; GFP not shown) and *Tg*(*lfabp*:dsRed;*elastaseA*:eGFP) embryos (L, N) stained for 2F11 (blue) and Prox1 (red) reveals that in wild type embryos 2F11 expression is heightened in the hepatopancreatic ducts at 48 hpf (arrowhead, K) and 4 dpf (arrowhead L). *hdac1* mutant embryos express 2F11 throughout the organ-forming region at 48 hpf (M), however expression is heightened in the duct at 4 dpf (N). In addition, wild type embryos express dsRed in the liver and GFP in the pancreas at 3 and 4 dpf (L) while only a subset of *hdac1* mutants express dsRed and GFP at 4 dpf (N).

The liver in *hdac1* mutant embryos is significantly smaller when compared to wild type siblings at 48 hpf (Figs. 2E, E′, I, I′). To determine if this phenotype is caused by either defects in liver specification, differentiation and/or growth, we investigated the expression of a set of genes involved in each of these processes(Field et al., 2003b). We assessed early stages of liver formation using the transgenic *Tg(gutGFP)*^*s854*^ line, expressing GFP throughout the developing endoderm (Field et al., 2003b), in conjunction with an antibody against Prox1. In wild type embryos, Prox1 is expressed by forming hepatoblasts and is present throughout the liver-forming region of the ventral endoderm at 24 hpf (Figs. 2B, B′; Ober et al., 2003). Prox1 expression is maintained within the forming liver bud (Figs. 2C, C′), and subsequently in differentiating hepatocytes (Figs. 2D, D′, E, E′). In *hdac1* mutant embryos, Prox1 expression is absent in the hepatic endoderm at 24 hpf (Figs. 2F, F′), and is not initiated until 28 hpf (Figs. 2G, G′). At this stage, it is expressed in the ventral endoderm in a pattern similar to the one observed in wild type siblings at 24 hpf, however reduced in cell number and intensity. To determine if this delay in expression is specific to Prox1 or is indicative of a defect in hepatoblast specification, we examined *hhex* expression. Similarly to Prox1, *hhex* is expressed in newly formed hepatoblasts (Liao et al., 2000; Ober et al., 2003). Consistent with the observed defects in Prox1 expression, *hhex* expression is absent in the liver-forming region of the endoderm in *hdac1* mutants at 24 hpf (Fig. 3C) and present in a reduced domain at 30 and 48 hpf (data not shown, Fig. 3D). In wild type embryos, the liver bud forms by hepatoblast aggregation while leftward growth occurs (Figs. 2C, C′). In contrast in *hdac1* mutant embryos, the hepatic cells first aggregate in an anteriomedial position and then grow towards the left side (Figs. 2H, H′, I, I′).

The observed delay in hepatoblast specification could be due to a reduction of mesodermally derived specification factors. Therefore, we examined *prometheus/wnt2bb (prt/wnt2bb)* expression. In wild type, *prt/wnt2bb* is expressed bilaterally in the LPM adjacent to the presumptive hepatic domain from around 18 hpf onwards, and has been shown to promote early liver formation (Ober et al., 2006). We observe reduced *prt/wnt2bb* expression at 26 hpf in *hdac1* mutants (Fig. 3F), likely accounting for the defect in the onset of hepatoblast specification.

To assess whether there is an additional defect in hepatocyte differentiation we examined the expression of *ceruloplasmin (cp)*, a plasma protein expressed in differentiating hepatocytes from around 36 hpf (Korzh et al., 2001). Absence of *cp* expression in *hdac1* mutants at 48 hpf (Fig. 3I) implies that hepatoblasts fail to differentiate into mature hepatocytes. Unlike in wild type, at 3 and 4 dpf *cp* expression can be detected in only 30% (*n* = 16) and 50% (*n* = 18) of *hdac1* mutant embryos, moreover the domain of expression is greatly reduced (Fig. 3J). We examined hepatocyte differentiation further using a transgenic reporter line expressing dsRed from 3 dpf onwards under the control of the *liver fatty acid binding protein (lfabp)* promoter in the liver (Fig. 3L; Dong et al., 2007). While at 3 dpf *hdac1* mutants fail to express dsRed (*n* = 18, data not shown), expression is detected in a subset of embryos at 4 dpf (Fig. 3L) and by 5 dpf in 45% of the embryos (*n* = 13; Fig. 3N), confirming hepatocyte differentiation is severely affected in these embryos.

The formation of the extrahepatopancreatic duct, connecting the liver and pancreas to the alimentary canal, is connected to forming a functional liver. Therefore, we analysed hepatopancreatic duct formation by investigating 2F11 expression between 40 hpf and 4 dpf (Dong et al., 2007). Initially, 2F11 is expressed throughout the forming liver, both pancreatic anlagen and the presumptive ductal tissue, and over time becomes restricted to the forming ducts (Figs. 3K, L). Similar to the onset of hepatic expression, we observe reduced 2F11 expression in *hdac1* mutant embryos when compared to their siblings at 40 hpf (data not shown; *n* = 8). However, at 48 hpf a strong expansion of the 2F11 expressing domain is observed in *hdac1* mutants (Fig. 3M), which appears to have failed to undergo morphogenesis when compared to wild type siblings (Fig. 3K). At 3 and 4 dpf in wild type, 2F11 expression is heightened in the extrahepatopancreatic ductal system (Fig. 3L; data not shown). Similarly, in *hdac1* mutants, we observed heightened 2F11 expression in the extrahepatopancreatic ductal tissue at 3 and 4 dpf (86% *n* = 6, 100% *n* = 9, respectively; data not shown; Fig. 3N). However, heightening of 2F11 in intrahepatic ducts fails to occur in *hdac1* mutants at 3 and 4 dpf (data not shown). Despite a generally wild type-like appearance of the extrahepatopancreatic ducts in *hdac1* mutant embryos at these stages, cellular and ductal morphology is altered and lumen formation appears impaired. Notably, 2F11 ductal expression in *hdac1* mutant embryos is not dependent on hepatic differentiation as indicated by absence of dsRed expression (data not shown).

Altogether, these data suggest that *hdac1* is crucial for the timely execution of liver specification and differentiation. Similarly, *hdac1* is required for the formation of the adjacent extrahepatopancreatic ducts. Strikingly, liver specification occurs, but is set back by approximately 6 h. However, the subsequent lag of at least 50 h in differentiation in *hdac1* mutants appears to be more severe.

### Hdac1 is cell-autonomously required for hepatocyte differentiation

During the initial stages of liver development *hdac1* is expressed in both endodermal and mesodermal tissues (Pillai et al., 2004; Yamaguchi et al., 2005; data not shown). During these stages, *prt/wnt2bb* expression in the LPM is reduced in *hdac1* mutants (Figs. 3E, F). To elucidate whether *hdac1* is required cell-autonomously within the endoderm to drive hepatic development, we performed mosaic analysis experiments. Wild type cells were labelled with rhodamine or fluorescein dextran to trace them after transplantation into *hdac1* mutant embryos. To convert cells to an endodermal fate or exclude them from this lineage, we manipulated levels of Casanova/Sox32 (Cas/Sox32) expression, the main endodermal determinant (Alexander et al., 1999). Wild type cells were co-injected with *cas/sox32* mRNA (Stafford et al., 2006) or *cas/sox32* morpholino oligonucleotides to direct them towards or exclude them from the endodermal lineage.

**Fig. 4:**
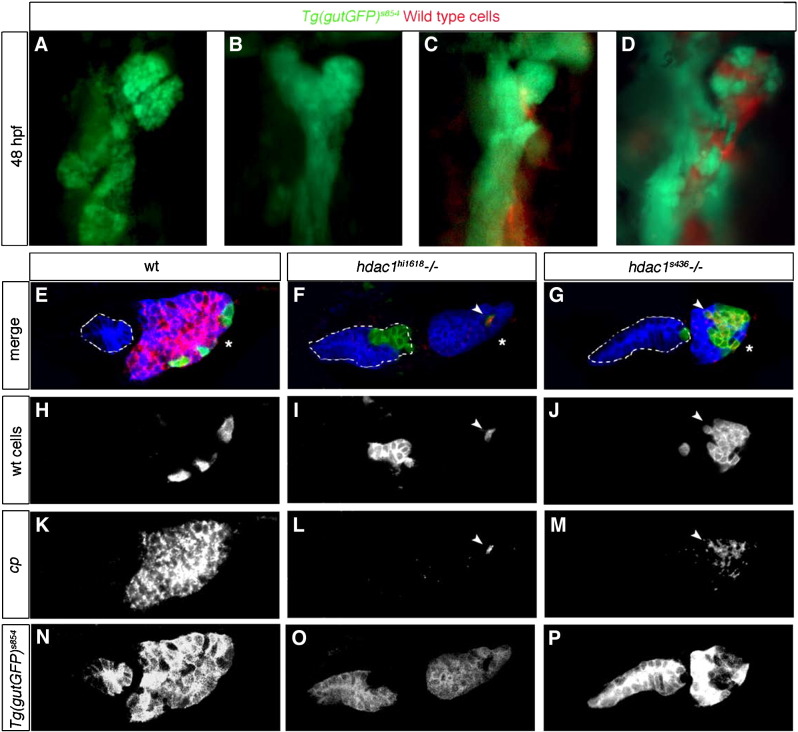
Cell-autonomous requirement for Hdac1 in hepatocyte differentiation. Ventral views of wild type (A) and *hdac1* mutant (B) organ-forming region at 48 hpf using the *Tg(gutGFP)*^*s854*^ line. (C, D) Morphological analysis of cell transplantation experiments; wild type cells (red) transplanted into *hdac1* mutants expressing the *Tg(gutGFP)*^*s854*^ transgene. (C) Wild type cells contributing to the mesoderm fail to rescue liver morphology in *hdac1* mutants. (D) Wild type cells contributing to the endoderm of *hdac1* mutants rescue liver morphology. (E–P) Cell transplantation experiments were analysed by fluorescent in situ hybridisation in conjunction with immunostaining in transverse sections at liver level, using the *Tg(gutGFP)*^*s854*^ line (blue) to highlight the endoderm, *cp* (red) to assess hepatocyte differentiation, and transplanted wild type cells labelled with fluorescein (green). (E, H, K, N) Wild type embryos express *cp* in the liver at 48 hpf. Wild type cells contributing to the hepatic endoderm of *hdac1* mutants express *cp* (arrowhead, F, I, L, G, J, M), however wild type cells contributing to the adjacent digestive tract fail to express *cp* (F, G). To validate that transplanted host cells injected with *cas* mRNA contribute to the endoderm, *Tg(gutGFP)*^*s854*^ embryos were used as donors in a subset of transplants (E, N, G, P); due to the mild mosaicism of the *Tg(gutGFP)*^*s854*^ expression, we observe in a subset of transplanted cells very low GFP expression. Asterisks indicate liver, white dashed lines outline the digestive tract.

At 48 hpf, analysis of liver morphology indicates that wild type cells contributing to the endoderm rescue liver bud morphology (14%, *n* = 11; Fig. 4D), whereas small groups of wild type cells contributing to the mesoderm fail to induce wild type-like liver bud formation in the endoderm (0% rescue; *n* = 7; Fig. 4C). However, we cannot rule out that the mesodermal clones are too small to cause an apparent rescue. Next, we examined *cp* expression, which is present in the liver of wild type embryos at 48 hpf (Fig. 4E). Analysis of wild type cells contributing to the hepatic endoderm of *hdac1* mutant embryos reveals that these cells express *cp* at 48 hpf (100%, in total *n* = 16; with *n* = 6 for *hdac1*^*s436*^ and *n* = 10 for *hdac1*^*hi1618*^, respectively; Figs. 4F, G), while the surrounding mutant cells fail to express *cp*. Wild type cells contributing to the liver express *cp* independent of the clone size, in small (Fig. 4F) and big clones (Fig. 4G). Wild type cells contributing to the mutant endoderm of the digestive tract are *cp* negative (100%, in total *n* = 24; with *n* = 14 for *hdac1*^*s436*^ and *n* = 12 for *hdac1*^*hi1618*^, respectively; Figs. 4F, G). These findings indicate a cell-autonomous requirement for *hdac1* within the endoderm in hepatic development.

### Histone deacetylases are required during late somitogenesis stages for liver specification

Hdac1 is widely expressed during embryonic development and is required for multiple processes of endodermal organogenesis. This raises the question as to whether the observed defects are specific to these later steps in organogenesis or are secondary to defects in early endoderm development. To determine the time of Hdac requirement during liver development, we used Trichostatin A (TSA), which inhibits class I and class II Hdacs (Yoshida et al., 1990). TSA was applied to wild type embryos at various time points. Digestive tract morphogenesis and liver specification and differentiation were subsequently examined by the pan-endodermally expressed transcription factor *foxA1* (Odenthal and Nusslein-Volhard, 1998), and by *hhex*, *prt/wnt2bb* and *cp* expression, respectively.

**Fig. 5:**
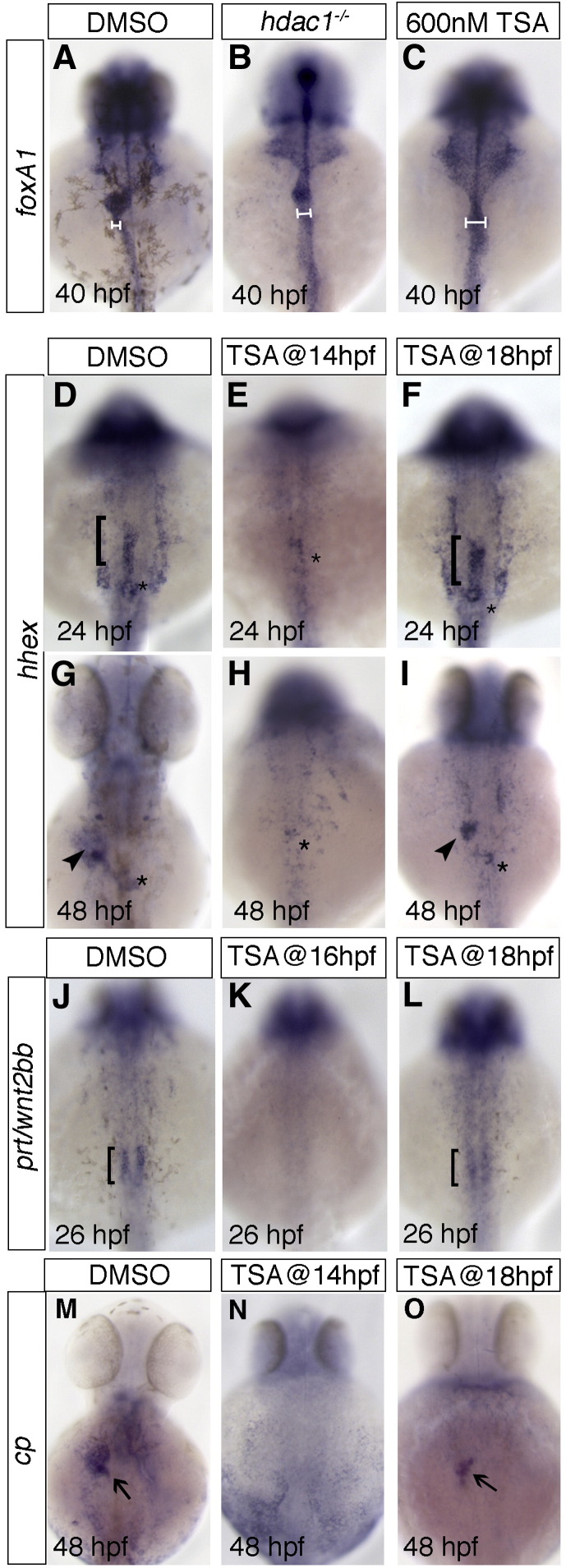
Histone deacetylase function is required during late somitogenesis for liver specification and differentiation. TSA was applied at various time points, and digestive tract morphogenesis, liver specification and differentiation were examined by *foxA1*, *hhex*, *prt/wnt2bb*, and *cp* expression, respectively. 600 nM TSA produced the closest phenocopy of the *hdac1*^*s436*^ phenotype when assessed by *foxA1* expression (compare panel B and C). This concentration was used in subsequent experiments. Horizontal bars show an expansion of the digestive tract in both TSA treated and *hdac1* mutants when compared to wild type embryos (compare panels B, C with A). (D–I) Hepatoblast specification in TSA treated embryos was assessed by *hhex* expression. Application of TSA at 14 hpf resulted in an absence of hepatic *hhex* expression at both 24 and 48 hpf (E, H). However, treatment at 18 hpf resulted in wild type expression of hepatic *hhex* at 24 hpf (bracket, F), although strongly reduced at 48 hpf (arrowhead, I). (J–L) *prt/wnt2bb* expression was assessed in TSA treated embryos. TSA treatment at 16 hpf resulted in an absence of *prt/wnt2bb* expression in the LPM abutting the organ-forming endoderm (K), however application of TSA at 18 hpf resulted in wild type-like expression of *prt/wnt2bb* (bracket, L). (M–O) Hepatocyte differentiation in TSA treated embryos was assessed by *cp* expression. Embryos treated with TSA at 14 hpf showed no *cp* expression at 48 hpf (N), however embryos treated at 18 hpf express *cp*, but in a reduced domain (arrow, O). DMSO was added as a control in all cases (A, D, G, J, M), asterisk indicates position of the pancreas. Anterior to the top.

Treatment with 600 nM TSA closely phenocopied *hdac1* defects when assessed by *foxA1* expression (Figs. 5B, C). To elucidate Hdac function in liver specification, TSA was applied at various time points, and *hhex* expression was examined at 24 and 48 hpf. Application of TSA at 14 hpf resulted in an absence of hepatic *hhex* expression at both 24 and 48 hpf (Figs. 5E, H). However, treatment at 18 hpf resulted in wild type expression of hepatic *hhex* at 24 and a reduced domain of hepatic *hhex* expression at 48 hpf (Figs. 5F, I). Similarly, TSA application at 14 and 16 hpf resulted in an absence of mesodermal *prt/wnt2bb* expression at 26 hpf (Figs. 5J, K; data not shown), while *prt/wnt2bb* expression was present in embryos treated at 18 hpf (Fig. 5L). These data support a requirement for Hdacs between 14 and 18 hpf in initiation of hepatic specification.

To determine the temporal requirement for Hdacs in hepatocyte differentiation, TSA treated embryos were scored for *cp* expression at 48 hpf. Embryos treated at 14 hpf showed no *cp* expression at 48 hpf (Fig. 5N), while the majority of embryos treated at 18 hpf express *cp*, although in a reduced domain (85% *n* = 47; Fig. 5O). This implies that the requirement for Hdac1 between 14 and 18 hpf in hepatocyte differentiation is tightly linked to hepatic specification.

In both cases, although treatment with TSA at 18 hpf results in correct timely expression of both hepatic specification and differentiation genes, the domain of expression of these genes at 48 hpf is reduced in size. Treatment of embryos with TSA as late as 30 hpf still results in a smaller domain of *hhex* and *cp* expression at 48 hpf (data not shown), suggesting that class I Hdacs play not only a role in timely development of hepatocytes, but additionally have a critical function in the growth of the liver (Figs. 5I, O). Furthermore, these findings indicate that the observed defects in *hdac1* mutants are not secondary to defects in early endoderm formation.

### Hdac1 function promotes cell proliferation in the endoderm

**Fig. 6:**
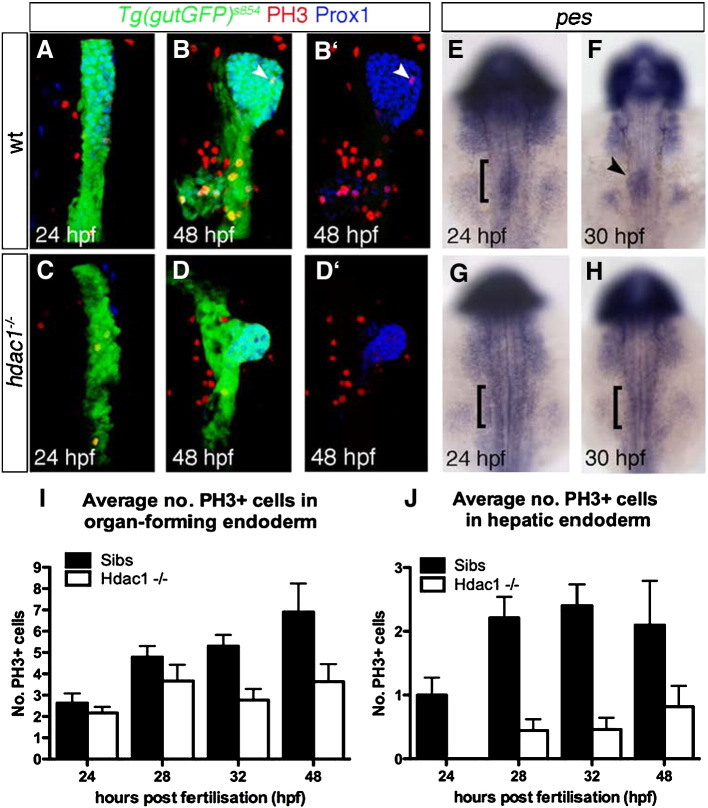
Loss of *hdac1* results in reduced cell proliferation in the organ-forming endoderm. (A–D′) Ventral projections using *Tg(gutGFP)*^*s854*^ line, stained for PH3-positive cells (red) and Prox1-positive hepatoblasts (blue) at 24 hpf (A, C), and 48 hpf (B, D); dorsal to the top. (E–H) In situ hybridisation analysis of *pes* expression. Wild type embryos express *pes* in the organ-forming region at 24 hpf (bracket, E) and hepatic region at 30 hpf (arrowhead, F). *hdac1* mutants lack *pes* expression in the organ-forming endoderm at both 24 and 30 hpf (brackets, G, H). (I, J) *hdac1* mutants display a decreased number of PH3-positive cells the endoderm and liver between 24 and 48 hpf. Numbers are supplied in Tables 1 and 2.

In *hdac1* mutant embryos at 48 hpf, liver size is severely reduced (Figs. 2E, E′, I, I′ and 3B, D). Moreover, Hdac1 has previously been implicated in regulating cell proliferation at different stages during mouse and zebrafish development (Cunliffe, 2004; Lagger et al., 2002; Stadler et al., 2005; Yamaguchi et al., 2005). We therefore investigated the role of *hdac1* in cell proliferation in the organ-forming endoderm using antiphospho-Histone H3 (PH3) staining to identify cells undergoing mitosis in sibling and *hdac1* mutant embryos. The organ-forming endoderm, comprises all endodermal cells marked by *Tg(gutGFP)*^*s854*^ expression from the anterior border of the hepatic domain to the posterior margin of the endocrine (24–32 hpf) or exocrine pancreas (48 hpf), respectively. We observed that the number of PH3-positive cells was reduced in *hdac1* mutant organ-forming endoderm between 24 and 48 hpf when compared with sibling endoderm (Figs. 6A–D; Table 1); initially, between 24 and 28 hpf, proliferation rates in *hdac1* mutant embryos are slightly reduced when compared with sibling embryos (19.2% and 22.9% reduction, respectively), but are severely reduced at 32 and 48 hpf (47.2% and 48.8% respectively; Fig. 6I). Importantly, cell proliferation decreases not only in the non-hepatic endoderm, but also in the hepatic endoderm (Table 2). Proliferation in hepatoblasts is reduced by 80% and 50% in *hdac1* mutants at 28–32 and 48 hpf respectively (Fig. 6J). This is in agreement with a smaller hepatic domain observed in wild type embryos treated with TSA at 18 hpf (Fig. 5O). At 24 hpf, proliferation in the presumptive hepatic cells could not be scored in *hdac1* mutant embryos as Prox1-positive cells were absent at that stage (Figs. 6C, J). However, it is possible that the reduced number of PH3-positive hepatoblasts is the consequence of a smaller hepatic primordium.

One candidate for regulating cell proliferation in *hdac1* mutant embryos is the gene *pescadillo* (*pes*; Allende et al., 1996). Its expression has been shown to be induced as hepatocytes enter the cell cycle (Lerch-Gaggl et al., 2002) and to promote cell cycle progression in yeast (Sakumoto et al., 2001). In wild type embryos, *pes* is expressed in the liver-forming domain at 24 hpf and onwards (Figs. 6E, F). In *hdac1* mutant embryos we fail to observe heightened *pes* expression in the hepatic domain, whereas it is up-regulated in the eyes, at 24 and 30 hpf (Figs. 6G, H; data not shown). The latter is in agreement with a previously reported increase in cell proliferation in the eye (Stadler et al., 2005; Yamaguchi et al., 2005). This confirms that reduced proliferation observed in the organ-forming endoderm is not due to a general reduction of proliferation in the entire embryo, suggesting that *hdac1* function depends on the tissue context. Importantly, *pes* expression levels in *hdac1* mutant endoderm appear wild type-like from 48 hpf onwards (data not shown).

Increased rates of cell death in the hepatic endoderm could contribute to the reduction of liver size observed in *hdac1* mutant embryos at 48 hpf. Therefore we examined cell death in *hdac1* mutant and sibling embryos at 30 and 48 hpf using Tunel staining. While changes were observed in other domains of the developing embryo, no obvious differences in cell death rates were observed in the organ-forming endoderm between *hdac1* mutant and sibling embryos, (Supplemental Fig. 3).

In summary, these data show a requirement for Hdac1 in promoting cell proliferation within the organ-forming endoderm between 24 and 48 hpf.

### Requirements for Hdac1 in endocrine pancreas formation and exocrine pancreas initiation

**Fig. 7:**
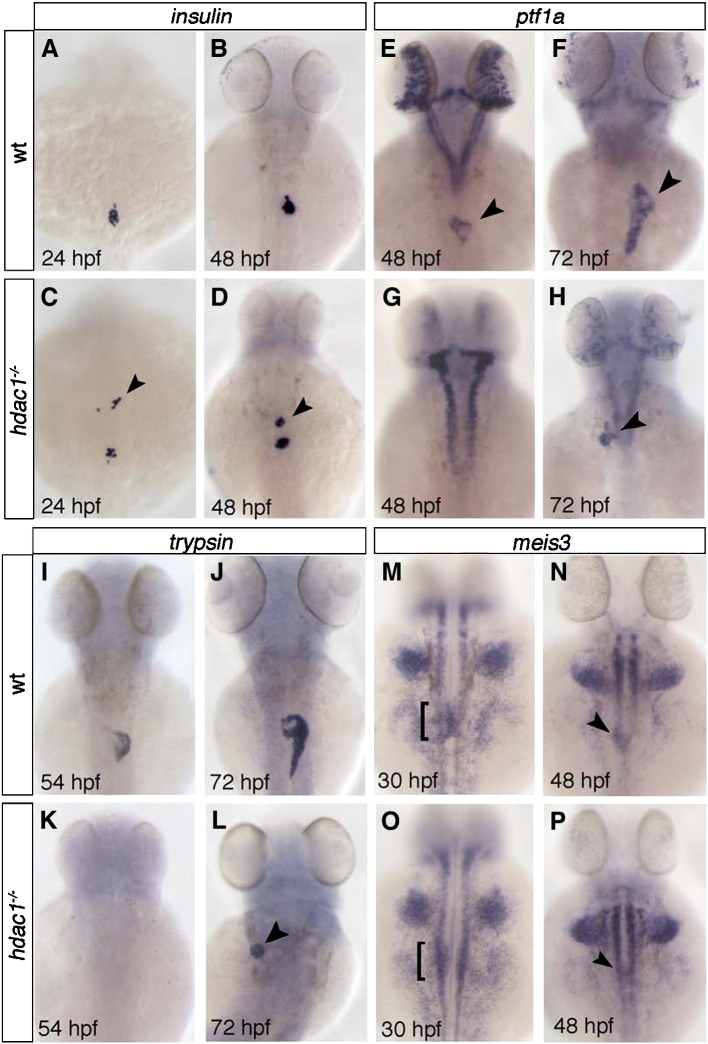
Hdac1 depletion results in ectopic endocrine tissue formation and defects in exocrine pancreas specification. (A–D) *insulin* is expressed in a single cluster of endocrine β-cells in wild type embryos at 24 and 48 hpf (A, B). *hdac1* mutants display ectopic anterior clusters of *insulin*-expressing cells at 24 and 48 hpf (arrowheads, C, D). (E–H) *ptf1a* is expressed in the exocrine pancreas at 48 and 72 hpf (arrowhead, E, F). *hdac1* mutant embryos lack pancreatic *ptf1a* expression at 48 hpf (G), but express *ptf1a* at 72 hpf in a reduced domain (arrowhead, H). (I–L) *trypsin* expression in the exocrine pancreas in wild type siblings at 54 hpf (I) is absent in *hdac1* mutants (K), however is detected in a subset of *hdac1* mutants at 72 hpf (arrowhead, L). (M–P) Wild type embryos express *meis3* medially in the LPM at 30 hpf (bracket, M) and 48 hpf (arrowhead, N). *hdac1* mutants display *meis3* expression bilateral to the endoderm at 30 hpf (bracket, O) and medially at 48 hpf (P).

The pancreas is derived from two primordia. In zebrafish, the endocrine pancreas arises from bilateral groups of cells aggregating to form the dorsal islet at 24 hpf (Biemar et al., 2001; Field et al., 2003a), subsequently, exocrine pancreas formation is initiated ventrally around 34 hpf (Field et al., 2003a). To assess pancreas formation, the expression of genes found in both the endocrine and exocrine pancreas was analysed. In wild type embryos, *insulin* is expressed by β-cells of the endocrine islet, from around 15 hpf onwards (Biemar et al., 2001). At 20 hpf, *insulin* expression in *hdac1* mutant and sibling embryos is indistinguishable, despite a variable delay observed at 18 hpf (data not shown). *insulin*-expressing endocrine cells aggregate into a single islet at the posterior end of the organ-forming region in wild type at 24 hpf (Figs. 7A, B). In contrast, in *hdac1* mutant embryos ectopic anterior groups of insulin-expressing cells are observed at 24 hpf (Fig. 7C) and are maintained at 48 hpf (Fig. 7D), as revealed by staining for *insulin* and Islet1/2 expression (Figs. 7D and 2G′, H′). These were observed in about 61% of *hdac1* mutants (*n* = 83) and in 20% of phenotypically wild type siblings (*n* = 245) at different stages. This indicates that Hdac1 plays a distinct role in the formation of the endocrine pancreas, most likely in a dosage dependent manner. At this stage we cannot distinguish whether this phenotype is caused by aberrant migration of endocrine cells or if these cells may have been incorrectly specified due to gain of promoting factors or loss of repressive activity within tissues anterior to the endocrine islet.

Analysis of the organ-forming region in *hdac1* mutants using the *Tg(gutGFP)*^*s854*^ line indicated that the exocrine pancreas failed to form at 34 hpf (Figs. 2H, H′) or at 48 hpf (Figs. 2I, I′). Therefore, we assessed embryos for expression of *ptf1a*, the earliest known gene to be detected in the exocrine pancreas (Lin et al., 2004; Zecchin et al., 2004), and *trypsin*, a marker of exocrine pancreatic function. In controls at 48 and 72 hpf, *ptf1a* is expressed in the exocrine pancreas (Figs. 7E, F). However in *hdac1* mutant embryos, expression cannot be detected at 48 hpf (Fig. 7G). At 72 hpf, *ptf1a* is expressed in the majority of *hdac1* mutant embryos, albeit in a reduced and often dysmorphic domain (90%, *n* = 11; Fig. 7H). Similarly, expression of *trypsin* is first detected around 72 hpf in a subset of *hdac1* mutant embryos (25%, *n* = 3; Fig. 7L). In about 65% of *hdac1* mutants expressing *ptf1a* or *trypsin* in the exocrine pancreas at 72 hpf, the expression domain appears to be placed left to the midline. Finally, we examined ventral pancreas formation using a transgenic reporter line expressing GFP under the control of the *elastaseA* promoter. While none of the *hdac1* mutant embryos expressed the reporter gene at 3 dpf, a subset of embryos were found to express GFP at 4 dpf (Fig. 3N), further supporting that Hdac1 is required for establishment of the exocrine pancreas. The lag of pancreas specification differs from the one observed in the liver, as it is initiated only in a subset of embryos about 30–40 h later than in wild type compared to an approximately six hour delay observed for the liver.

Meis3, a TALE-box protein, is required for both endocrine and exocrine pancreas development (diIorio et al., 2007; Manfroid et al., 2007). Similar to *hdac1* mutants, embryos lacking *meis3* function display ectopic anterior patches of *insulin* expression (diIorio et al., 2007), as well as defects in ventral pancreas formation (Manfroid et al., 2007). To assess whether loss of *meis3* could account for the endocrine pancreas phenotype observed in *hdac1* mutants, we examined *meis3* expression in *hdac1* mutant embryos. In wild type, *meis3* is expressed bilaterally in the pancreatic LPM, anterior to the endocrine pancreas at 24 hpf, and medially as a result of LPM migration from 30 hpf onwards (Figs. 7M, N; Manfroid et al., 2007). In *hdac1* mutant embryos, bilateral *meis3* expression is present, though medial LPM migration fails to occur (100%, *n* = 18; Fig. 7O, Supplemental Fig. 2F). Medial *meis3* expression is detectable only in a subset of mutant embryos at 48 hpf (82%, *n* = 27; Fig. 7P). This suggests that Hdac1 does not regulate *meis3* expression; however, it raises the possibility that both may interact on a protein level, to regulate common downstream targets.

The laterally displaced *meis3* expression domains observed in *hdac1* mutants at 30 hpf suggest a defect in medial migration of the left and right LPM in the organ-forming domain. The latter might in addition cause a mild gut-looping defect in *hdac1* mutant embryos apparent at 48 hpf (Figs. 2I, I′). It has previously been shown that the leftward looping of the gut, which is initiated in wild type around 30 hpf, is the result of asymmetric movements of the LPM (Horne-Badovinac et al., 2003). During this process, the left LPM migrates dorsal to the gut across the midline, while the right LPM moves down ventrally (Supplemental Figs. 1A, B). We find in transverse sections in the organ-forming region of *hdac1* mutant embryos that both the left and the right LPM failed to move medially at 30 hpf (*n* = 30; Supplemental Figs. 1C, D). These findings suggest that the loss of medial *meis3* expression in the LPM (Figs. 7O, P) as well as the mild gut-looping defect are possibly due to LPM migration defects.

Taken together, these findings reveal that Hdac1 plays distinct roles in the formation of both pancreatic lineages.

### Hdac1 promotes hepatic and exocrine pancreatic primordia formation at the expense of foregut tissue

**Fig. 8:**
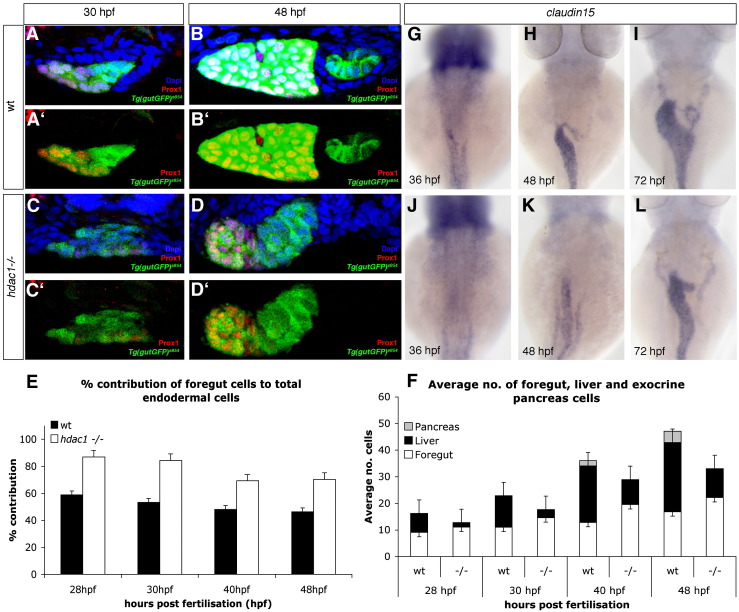
Loss of Hdac1 function results in an increase of non-hepatic foregut endoderm. (A–D) Transverse sections taken through the level of the liver, at 30 hpf and 48 hpf in wt (A–B′) and *hdac1* mutants (C–D′). To count endodermal cells, *Tg(gutGFP)*^*s854*^ embryos were stained for Prox1 (red), highlighting hepatoblasts, and Dapi (blue). (E) *hdac1* mutants display an increased percentage contribution of foregut cells to total endodermal cells between 28 hpf and 48 hpf when compared to wild type siblings. (F) *hdac1* mutant embryos have an increased number of foregut cells compared to wild type embryos between 28 and 48 hpf (white bars). However, *hdac1* mutant embryos display an overall reduction in number of total endodermal cells when compared to wild type embryos (compare total bar height). (E, F) Number of wild type embryos analysed: 28 hpf *n* = 12, 30 hpf *n* = 14, 40 hpf *n* = 13, 48 hpf *n* = 13. Number of *hdac1* mutants analysed: 28 hpf *n* = 10, 30 hpf *n* = 11, 40 hpf *n* = 10, 48 hpf *n* = 10. (G–L) *hdac1* is required for correct temporal expression of *claudin15*. Wild type embryos express *claudin15* in the endoderm from 36 hpf onwards (G–I), whereas *hdac1* mutants fail to express *claudin15* at 36 hpf (J), however, expression is detected at 48 hpf (K) and 72 hpf.

In addition to a significantly reduced liver bud, we observed an increase of foregut tissue adjacent to the forming liver and pancreatic primordium in *hdac1* mutant embryos compared to sibling embryos between 40–48 hpf (Figs. 2D, E, H, I; 4E–G; 5A–C). To examine the possibility that defects in hepatic and exocrine pancreatic primordia formation leads to an expansion of alimentary canal tissue within the foregut domain, we determined the number of hepatic, pancreatic and gut cells in sections of the organ-forming endoderm at four stages between 28 and 48 hpf in *hdac1* mutant and sibling embryos (Figs. 8A–F). We observe a strong reduction of hepatic cell numbers and an absence of exocrine pancreatic tissue in *hdac1* mutants at the stages examined (Fig. 8F). Comparing the number of foregut cells to the total number of endodermal cells in a section in mutant and sibling embryos, we observe a 21.2 to 31% increase of foregut endodermal cells in *hdac1* mutant embryos between 28 and 48 hpf (wt siblings: 28 hpf = 58.9%, 30 hpf = 53.3%, 40 hpf = 48.1%, 48 hpf = 46.3%; *hdac1* mutants: 28 hpf = 86.8%, 30 hpf = 84.2%, 40 hpf = 69.3%, 48 hpf = 70.2%; Fig. 8E). These changes are statistically significant: 28 hpf *p* = 0.011, 30 hpf *p* = 0.0002, 40 hpf *p* < 0.0001, 48 hpf *p* = 0.001. These findings suggest that Hdac1 is required for timely formation of the hepatic and pancreatic primordia from the foregut endoderm and in the absence of *hdac1* function liver and pancreas formation are not only delayed, but in addition occur in a smaller number of cells, resulting in an increase of neighboring foregut tissue.

Furthermore, these experiments have revealed an overall reduction of endodermal cells in the organ-forming region in *hdac1* mutants between 28 and 48 hpf, (28 hpf = 21.5%, 30 hpf = 22.3%, 40 hpf = 19.9%, 48 hpf = 30%; Fig. 8F), confirming the results of the proliferation studies (Fig. 6).

In parallel, we have examined *claudin15* expression, which is required for lumen formation during gut development (Bagnat et al., 2007), to elucidate, whether loss of *hdac1* not only leads to an increase of foregut endoderm, but also promotes its subsequent differentiation. Analysis at 36 and 48 hpf revealed that the onset of *claudin15* expression is mildly delayed and the expression weaker at 48 hpf in *hdac1* mutant embryos, when compared to wild type siblings (0% *n* = 10 for 36 hpf, 91% *n* = 33 for 48 hpf; Figs. 8G, J, H, K). At 72 hpf, the gut appears thinner in *hdac1*^*s436*^ mutant embryos when compared to wild type siblings (*n* = 17; Figs. 8I, L), consistent with our observations in the *Tg(gutGFP)*^*s854*^ line at this and later stages (data not shown; Figs. 3L, N). Moreover, *hdac1* mutant embryos appear to exhibit an increase in 2F11-expressing enterocytes in the posterior foregut (Fig. 3N). However, as the overall shape of the alimentary canal in *hdac1* mutants at this stage appears slimmer, detailed analysis will be required to determine whether this is due to changes in cell morphology or proliferation, and similarly, whether the possible increase in enterocyte number is related to this.

Taken together, these data suggest that Hdac1 is required for establishing hepatic and exocrine pancreatic fates at the expense of non-hepatic foregut endoderm. In addition, Hdac1 appears to be required for the timely onset of digestive tract differentiation.

## Discussion

Complex multi-organ systems, such as the digestive system, in which the liver, exocrine and endocrine pancreas arise from the foregut endoderm, demand tightly coordinated steps of patterning and morphogenesis to allow formation of each organ. As we are beginning to identify the roles of several transcription factors within these processes, we need to understand how their activities are regulated. Here, we present genetic evidence that the broadly expressed chromatin remodelling factor Hdac1 has multiple distinct roles in liver, pancreas and foregut development.

### Specificity of Hdac1 in endodermal organogenesis

Hdac1 has been shown to be required for numerous processes during embryonic development in *Caenorhabditis elegans*, *Drosophila*, zebrafish and mice (Calvo et al., 2001; Cunliffe, 2004; de Ruijter et al., 2003; Dufourcq et al., 2002; Lagger et al., 2002; Mannervik and Levine, 1999; Pillai et al., 2004). This raises the question whether the observed defects in endodermal organogenesis in *hdac1* mutants are specific or secondary to general defects in early endoderm formation. Three lines of evidence support the view that Hdac1 carries out distinct functions: first, we detect no obvious changes in early endodermal *sox17*, *her5* and *foxA1* expression during late gastrulation stages and at 24 hpf, respectively (data not shown). *hdac1* is ubiquitously expressed from the 1-cell stage until 18 hpf (Cunliffe, 2004; Nambiar et al., 2007; Pillai et al., 2004; Yamaguchi et al., 2005), suggesting contribution of maternal wild type Hdac1 may thus allow early endodermal development to proceed undisrupted. Second, inhibition of Hdac activity between 14 and 18 hpf by application of TSA leads to a severe or complete loss of hepatoblast specification, confirming that this defect is specific to Hdac1 function at this stage and not secondary to earlier defects in endoderm development. Concomitantly, we found that expression of *prt/wnt2bb*, known to promote hepatoblast formation, is absent under these conditions. Third, the different fates developing from the foregut endoderm appear affected differently — liver and exocrine pancreas specification and differentiation are impaired, endocrine islet and extrahepatopancreatic duct formation are disrupted and foregut tissue is initially expanded.

### Hdac1 functions in hepatic and pancreatic organogenesis

Our genetic study indicates that *hdac1* is required for the timely onset of hepatoblast specification and their subsequent differentiation. Analyses of *hhex* and Prox1 expression in *hdac1* mutant embryos as well as embryos in which Hdac function was blocked by application of TSA, reveal defects in the onset of hepatoblast formation. Timed inhibition of Hdac activity (preferentially class I) using Valproic acid revealed similar defects in early liver development (Farooq et al., 2008). In *hdac1* mutants, *prt/wnt2bb* expression in the LPM is reduced, possibly leading to the delay in hepatoblast formation. Moreover, inhibition of Hdac activity by TSA treatment reveals a requirement for Hdac activity before 18 hpf, which coincides with *prt/wnt2bb* expression in the LPM (Ober et al., 2006). Together this supports the view that decreased *prt/wnt2bb* levels are the underlying cause for the delay in hepatoblast formation in Hdac1 depleted embryos. Hdac1 activity may regulate – directly or indirectly – levels of *prt/wnt2bb* expression in the LPM. Importantly, although we observe LPM migration defects at later stages, the distance between the bilateral *prt/wnt2bb*-expression domains in the LPM appears not altered when compared to wild type at the time of hepatoblast formation, indicating that LPM migration defects occur at subsequent stages. It is intriguing to speculate that Hdac1 controls signals from the endoderm that are required to promote *prt/wnt2bb* expression in the mesoderm, similar to the role of the endoderm in inducing heart development (Foley et al., 2006). Likewise, knock-down of Hhex or Pdx1 function in zebrafish results in organ-laterality defects, suggesting a cross-talk between the organ-forming endoderm and neighboring mesoderm (Wallace et al., 2001; Yee et al., 2001).

We used mosaic analyses to demonstrate that Hdac1 cell-autonomously regulates hepatic development, showing a requirement for Hdac1 function within the endoderm. This is in line with findings demonstrating that isoforms of the intrinsic hepatic differentiation factor HNF4α interact with Hdac complexes to mediate hepatocyte maturation in vitro (Torres-Padilla et al., 2002). Moreover, our data indicate that Hdac1 regulates the early hepatic expression of the BRCT-domain containing factor Pes. Pes has been implicated in linking cell growth and cell division in yeast (Du and Stillman, 2002). In mice the highest *pes* mRNA expression levels have been detected in the liver (Lerch-Gaggl et al., 2002). In zebrafish *pes* mutants, the liver, pancreas and gut fail to expand (Allende et al., 1996). However, we observe wild type-like *pes* expression levels in all *hdac1* mutants at later stages as well as expression of hepatic differentiation genes in about 50% of *hdac1* mutants, suggesting that additional Hdacs are required for regulation of hepatic gene expression. Recent work has implicated an additive requirement for Hdac1 and Hdac3 in early hepatic development (Farooq et al., 2008), thus suggesting both factors complement each other's function.

Very little is known about the role of Hdacs in exocrine and endocrine pancreas development. Recent work in zebrafish has implicated complex interactions between the pancreatic endoderm and the adjacent LPM in exocrine pancreas specification and outgrowth (Manfroid et al., 2007). Similar interactions have been described in chicken and mouse for the formation of the dorsal and ventral pancreatic anlagen, which give rise to endocrine and exocrine tissue (Cano et al., 2007). Furthermore, requirements for Fgf signalling in these processes have been described (Cano et al., 2007; Manfroid et al., 2007). In zebrafish, loss of Fgf24 function leads to reduced or absent *meis3* and *isl1* expression in the neighboring LPM and impaired exocrine pancreas formation, while loss of Fgf24 and Fgf10 results in severely reduced or absent exocrine pancreata (Manfroid et al., 2007). While *meis3* expression levels in the LPM appear unaltered in *hdac1* mutants, the LPM is misplaced at 30 hpf. A requirement for Hdac activity in mediating Fgf signalling has been shown in *Xenopus* mesoderm induction and zebrafish development (Plaster et al., 2007; Xu et al., 2000). It will thus be interesting to examine additional potential interactions between Fgf signalling and Hdac1 function with regards to the establishment of the exocrine pancreas.

The fact that pancreas specification occurs with a delay of at least 30–40 h, suggests that the endoderm either maintains or gains the competence to respond to delayed exocrine pancreas specification in *hdac1* mutants. This is reminiscent of recent findings in hepatic development, where transient inhibition of Fgf and Bmp signalling in zebrafish delayed hepatoblast formation and differentiation for about 16 h, suggesting that the endoderm maintains it competence to respond to specification and differentiation signals (Shin et al., 2007).

In addition, Meis3 has been implicated in exocrine pancreas outgrowth, raising the intriguing possibility that Meis3 in the LPM promotes exocrine pancreas outgrowth (Manfroid et al., 2007). Therefore, it is possible that the LPM, which is misplaced in *hdac1* mutants at the time of pancreas specification, causes the altered location of the exocrine pancreas observed in a subset of mutant embryos.

Interestingly, we find that establishment of the scattered endocrine pancreatic cells at 20 hpf appears unaffected in *hdac1* mutant embryos, suggesting that this process is either Hdac1 independent or maternal contribution of wild type Hdac1 is sufficient to ensure its initial development. However, at 24 hpf ectopic clusters of endocrine pancreatic tissue are detected in *hdac1* mutant embryos anterior to the main islet. This finding suggests that Hdac1 is required for islet aggregation, either within in islet cells or in neighboring tissues promoting this process. Alternatively, Hdac1 may repress ectopic endocrine cell formation in endodermal tissue anterior to the main islet. It is interesting to note that increased RA signalling (Stafford and Prince, 2002) or inhibition of Hedgehog (Hh) signalling (diIorio et al., 2002) can lead to a comparable phenotype, with the latter appearing qualitatively more similar to the *hdac1* phenotype. In the zebrafish hindbrain, Hdac1 is required in neural precursor cells to maintain their responsiveness to Hh signalling (Cunliffe, 2004). Therefore, it is possible that interactions between Hdac1 and Hh and/or RA signalling are required for endocrine islet formation.

Glucose-regulated interactions between Hdac1/2, Pdx1 and/or Sox6 have been suggested to control *insulin* gene expression in mouse insulinoma cell lines (Iguchi et al., 2007; Mosley and Ozcan, 2004). However, our results suggest that initiation and maintenance of *insulin* expression in embryonic β-cells is independent of Hdac1 function until 48 hpf. Future studies are necessary to elucidate whether Hdac1 interacts with Pdx1 during pancreatic development and to determine additional partners and targets.

Interactions between the endoderm and the neighboring mesoderm are crucial for numerous steps in digestive system development (Grapin-Botton, 2005). It is possible that the LPM migration defects observed in *hdac1* mutant embryos are based on changes of the extracellular environment. A global gene expression profiling study carried out to identify targets of Hda-1, the Hdac1 homolog in *C. elegans*, identified extracellular matrix (ECM) related genes as major target genes (Whetstine et al., 2005). Also, in zebrafish timely migration and epithelialisation of the myocardial precursors, an anterior LPM derivative has been shown to require deposition of the ECM component Fibronectin (Trinh and Stainier, 2004). Furthermore, changes in ECM composition could be responsible for a possible defect in islet and extrahepatopancreatic duct morphogenesis.

Intriguingly, our findings show an increase in tissue of the alimentary canal in the area of the foregut and subsequently a mild delay in the onset of its differentiation. Analysis of mice organ cultures revealed that loss of intestinal Hdac (class I) activity leads to increased expression of differentiation genes (Tou et al., 2004). Moreover, cell-culture experiments show that TSA-mediated hyperacetylation promotes enterocyte differentiation (Archer et al., 2001). It will therefore be important to examine the temporal differentiation status of the alimentary canal on a cellular level.

In summary, our data demonstrate that Hdac1 promotes liver and exocrine pancreas development at the expense of the formation of foregut fates, suggesting a model in which a fate switch at the organ level is mediated by an epigenetic enzyme.

### Hdac mode of action—specificity versus redundancy

It is generally thought that class I Hdacs have more ubiquitous functions, while class II Hdacs act in a more tissue specific manner. Thus, it is surprising that we observe such distinct phenotypes. The fact that TSA treatment leads to more severe defects in hepatoblast specification than observed in *hdac1* mutant embryos implies that other class I or class II Hdacs are involved in this process. Several Hdacs may thus be required for liver formation, acting in parallel and/or sequentially, as has been suggested for osteoblast differentiation (Westendorf, 2007). Interestingly, loss of Hdac1 in mouse ES cells leads to an increase of Hdac2 and Hdac3 expression, however total histone deacetylase activity was significantly reduced (Lagger et al., 2002). In support of this possibility, recent work in zebrafish indicates that depletion of Hdac3 function leads to defects in liver specification and subsequent differentiation and that loss of both Hdac1 and Hdac3 function leads to more severe defects (Farooq et al., 2008). Similarly, depletion of DNA methyltransferase I and/or H3K9 histone methyltransferase Suv39h1 in embryos leads to organ-specific defects in terminal differentiation of the intestine, exocrine pancreas and retina (Rai et al., 2006).

Furthermore, additional Hdacs could act in a partially redundant manner together with Hdac1. This has been observed in tissue specific deletions of Hdac1 and Hdac2 in the developing myocardium where loss of either function has no obvious effect on cardiac development or function. However, cardiac specific deletion of both genes leads to defects in growth and differentiation (Montgomery et al., 2007). Hence, multiple Hdacs could act redundantly in hepatoblast specification, though the degree of this redundancy might vary. It is possible that Hdac1 interacts with other Hdacs in endodermal organogenesis, potentially in an organ and process specific manner.

In summary, our genetic studies reveal that Hdac1 is differently required for the formation of endodermal organs developing in close temporal and spatial proximity. Our findings in conjunction with other studies (Farooq et al., 2008; Rai et al., 2006) suggest that zebrafish represent a powerful model to investigate the roles of chromatin remodelling factors in controlling organ-specific gene expression programmes. Future work will have to determine the underlying molecular mechanisms and direct targets of Hdac1 in the development of the different organs.

## Figures and Tables

**Table 1:** Average number of PH3-positive cells in organ-forming endoderm

	hpf	24	28	32	48
*Siblings*	Av.	2.63	4.79	5.30	6.50
	s.d.	1.82	2.25	2.39	4.23
	*N*	16	19	20	10
*hdac1−/−*	Av.	2.17	3.75	2.77	3.64
	s.d.	1.20	2.29	1.88	2.73
	*N*	18	9	13	11

Av. Average, s.d. standard deviation, *N* number of embryos, hpf hours post-fertilisation. Proliferation rates are reduced in the organ-forming endoderm in *hdac1* mutants between 24 and 48 hpf.

**Table 2:** Average number of PH3-positive cells in hepatic endoderm

	hpf	24	28	32	48
*Siblings*	Av.	1	2.21	2.40	2.10
	s.d.	1.10	1.44	1.50	2.18
	*N*	16	19	20	10
*hdac1−/−*	Av.	0	0.44	0.46	0.82
	s.d.	0	0.53	0.66	1.08
	*N*	18	9	13	11

Av. Average, s.d. standard deviation, *N* number of embryos, hpf hours post-fertilisation. Proliferation rates are reduced in the hepatic endoderm in *hdac1* mutants between 24 and 48 hpf.
