# Supplementary figures and images for: Organ-specific requirements for Hdac1 in liver and pancreas formation
Source: Dev Biol. 2008 Oct 15;322(2):237–50. doi: 10.1016/j.ydbio.2008.06.040 (PMC3710974; doi:10.1016/j.ydbio.2008.06.040)

Supplemental Figure1 Noel et al

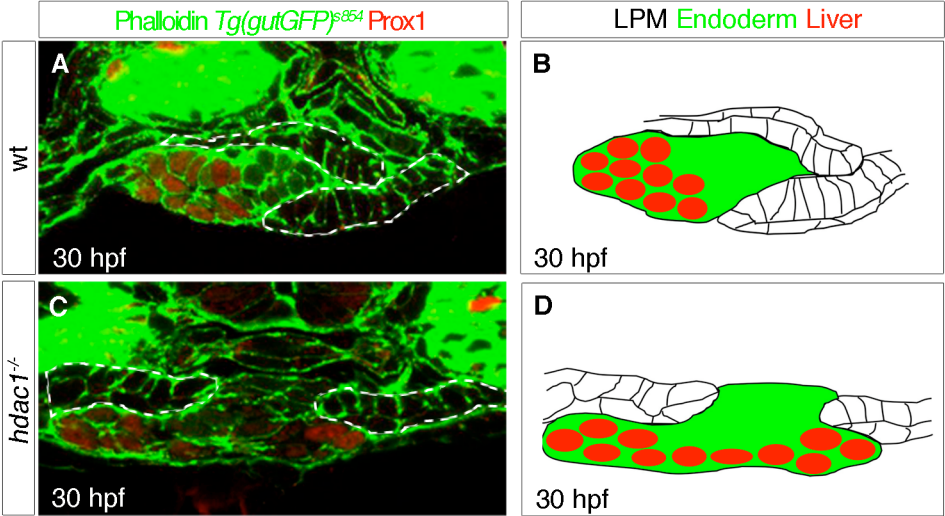

Supplement: Supplementary file 1 [file mmc1.pdf]

Supplemental Figure 2 Noel et al

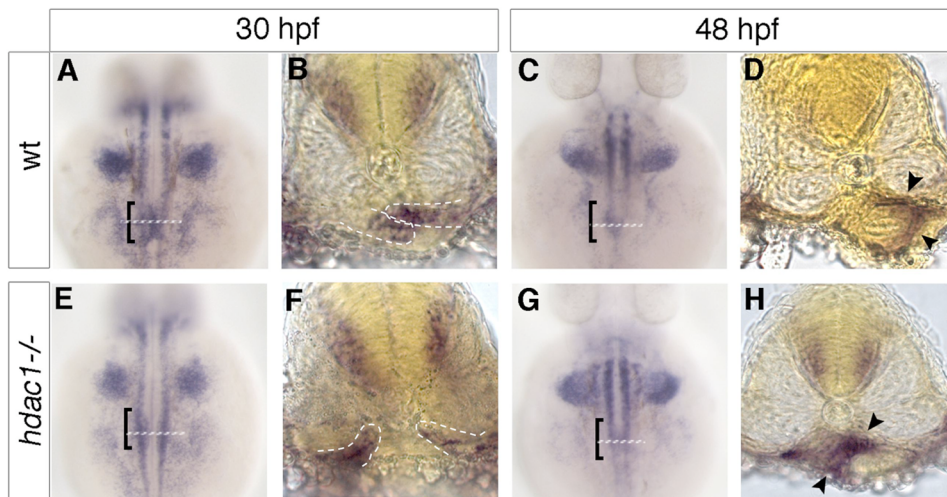

Supplement: Supplementary file 2 [file mmc2.pdf]

Supplemental Figure 3 Noel et al

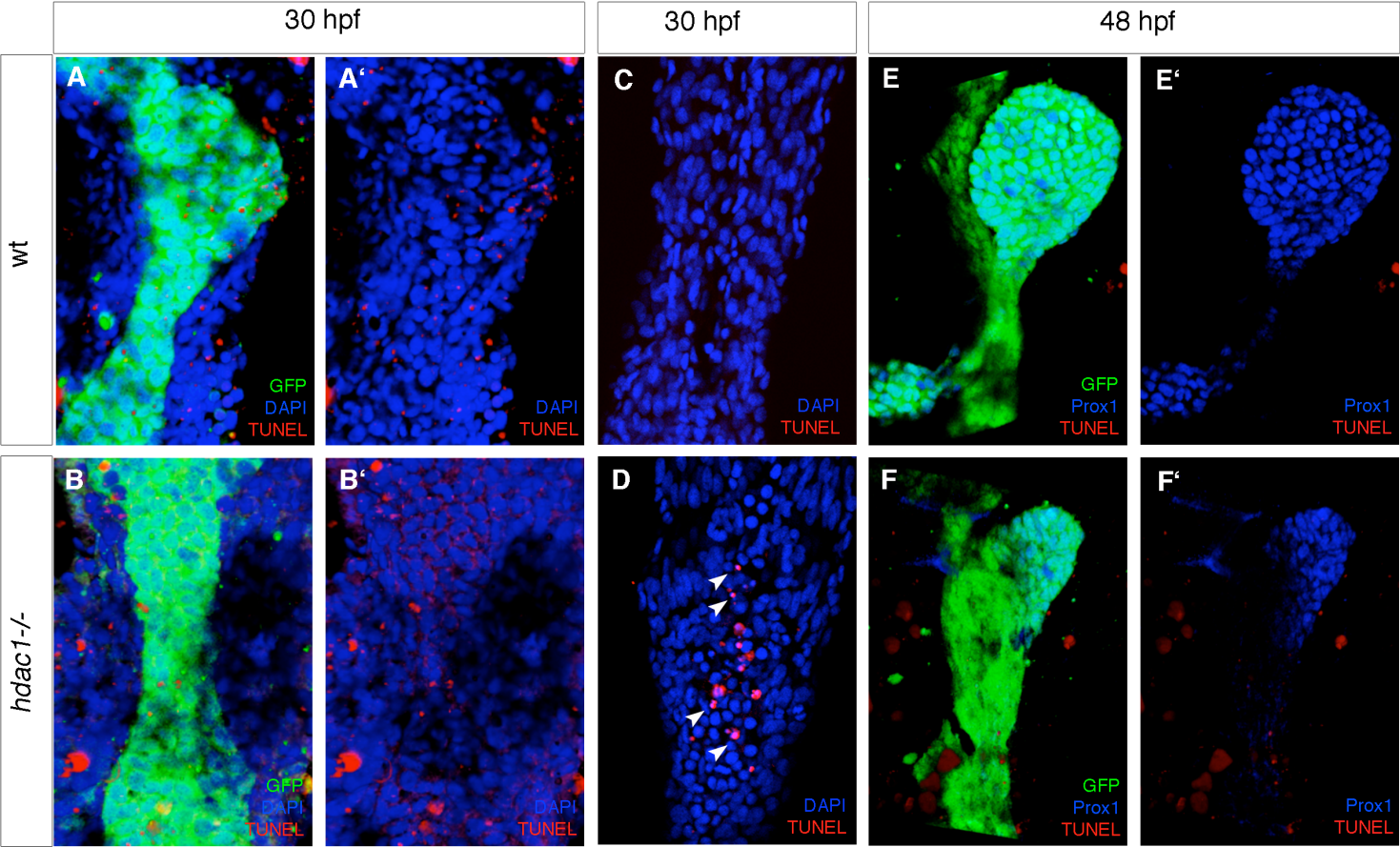

Supplement: Supplementary file 3 [file mmc3.pdf]
