# Supplementary material for: Organ-specific requirements for Hdac1 in liver and pancreas formation
Source: Dev Biol. 2008 Oct 15;322(2):237–50. doi: 10.1016/j.ydbio.2008.06.040 (PMC3710974; doi:10.1016/j.ydbio.2008.06.040)
Supplement: Supplementary file 4 [file mmc4.pdf]

Comparison of hepatic and pancreatic gene expression in the *hdac1*^{hi1618} and *hdac1*^{s436} alleles

		hdac1^{hi1618}	*hdac1*^{s436}
hhex	24 hpf	0% (n=17)	0% (n=17)
hepatoblast domain	30 hpf	87% (n=15)	88% (n=33)
cp	48 hpf	0% (n=23)	0% (n=15)
liver	72 hpf	6% (n=17)	19% (n=16)
	96 hpf	17% (n=23)	50% (n=18)
pitf1a	48 hpf	78% (n=32)	10% (n=20)
exocrine pancreas	72 hpf	100% (n=12)	100% (n=10)
insulin	24 hpf	0% (n=14)	39% (n=33)
single endocrine	30 hpf	19% (n=21)	31% (n=39)
pancreas	48 hpf	43% (n=14)	28% (n=44)
